# Pulmonary expanded polytetrafluoroethylene conduits with a hand-sewn tricuspid valve

**DOI:** 10.1093/icvts/ivaf020

**Published:** 2025-02-06

**Authors:** Shunsuke Matsushima, Ryota Takahashi, Sara Kubo, Akihiko Higashida, Yoshihiro Oshima, Hironori Matsuhisa

**Affiliations:** Department of Cardiovascular Surgery, Kobe Children’s Hospital, Kobe, Japan; Department of Cardiovascular Surgery, Kobe Children’s Hospital, Kobe, Japan; Department of Cardiovascular Surgery, Kobe Children’s Hospital, Kobe, Japan; Department of Cardiovascular Surgery, Kobe Children’s Hospital, Kobe, Japan; Department of Cardiovascular Surgery, Kobe Children’s Hospital, Kobe, Japan; Department of Cardiovascular Surgery, Kobe Children’s Hospital, Kobe, Japan

**Keywords:** right ventricular outflow tract reconstruction, pulmonary valve replacement, expanded polytetrafluoroethylene conduit, hand-sewn valve, pediatric patient, congenital heart disease

## Abstract

**OBJECTIVES:**

The biocompatibility of expanded polytetrafluoroethylene in the pulmonary position seems better than allogenic or xenogeneic reactivity. This study reviewed the application of pulmonary expanded polytetrafluoroethylene conduits having a hand-sewn tricuspid valve with diameters of 18–24 mm.

**METHODS:**

All patients receiving this conduit between 2010 and 2022 were evaluated. A 0.1-mm-thick membrane and a standard-wall tube of expanded polytetrafluoroethylene were used for cusp and conduit material, respectively.

**RESULTS:**

Eighty-four consecutive patients were included. The median operative age and weight were 12 (range, 1.2–40) years and 34 (range, 9.1–82) kg, respectively. Eighteen-, 20-, 22- and 24-mm conduits were used in 19, 5, 3 and 57 patients, respectively. The overall survival was 94% at 5 and 10 years with four non-valve-related deaths. There were five conduit replacements, all for 18-mm conduit stenosis. Freedom from conduit replacement was 98% and 83% at 5 and 10 years, respectively. Freedom from conduit stenosis ≥ moderate was 83% and 54% at 5 and 10 years, respectively. Freedom from pulmonary regurgitation ≥ moderate was 98% at 5 and 10 years. Linear mixed-effects models with echocardiographic data implied that 24-mm conduits functioned with a peak velocity <3.0 m/s and without moderate/severe regurgitation in patients with a body weight of up to 75 kg and a body surface area of up to 2.0 m^2^ for >12 years postoperatively.

**CONCLUSIONS:**

This conduit has shown favourable clinical outcomes and is a valid alternative, especially in young patients with increased risk for early failure of the existing products.

## Graphical abstract

**Figure:**
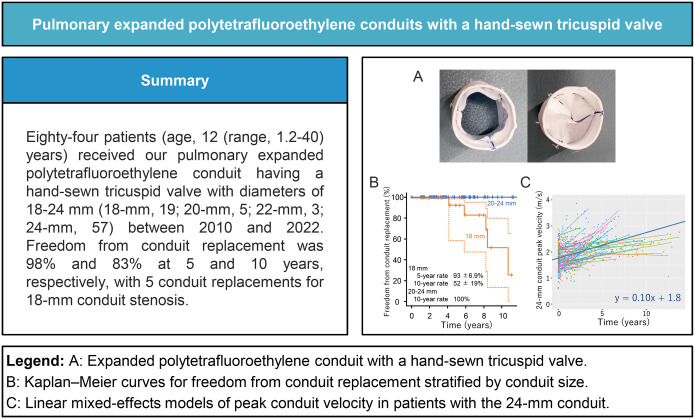

## INTRODUCTION

Right ventricular outflow tract (RVOT) reconstruction is essential for repairing various congenital heart defects. If the integrity of pulmonary valve structure is not well preserved or a valved conduit is used to establish right ventricle-to-pulmonary artery (RV-PA) continuity, subsequent valve insertion for pulmonary valve dysfunction is inevitable. Homografts and bioprostheses are often used [1–4], and it is hoped that these valve substitutes will provide competent valve function and adequate durability because both multiple sternotomies and short intervals from previous sternotomy are great risk factors for poor clinical outcomes [5]. Regarding their clinical application, however, there is still room for improvement, especially in the paediatric population [1–4].

Various initiatives have been done to develop an alternative superior to the existing products. Historically in Japan, where both homografts and bovine jugular vein conduits were unavailable, expanded polytetrafluoroethylene (ePTFE) has emerged as the material of choice for valved conduits [6–9]. The biocompatibility of ePTFE seems better than allogenic or xenogeneic reactivity, especially in young patients. A handmade ePTFE conduit with sinuses and cusps like normal semilunar valve’s structures showed excellent valvular function and improved durability [7]. However, their conduits require special manufacturing, such as thermal processing, leading to their limited global use [6]. In the USA, an ePTFE valved conduit with simpler designs are preliminarily applied in several centres [10].

**Figure 1:**
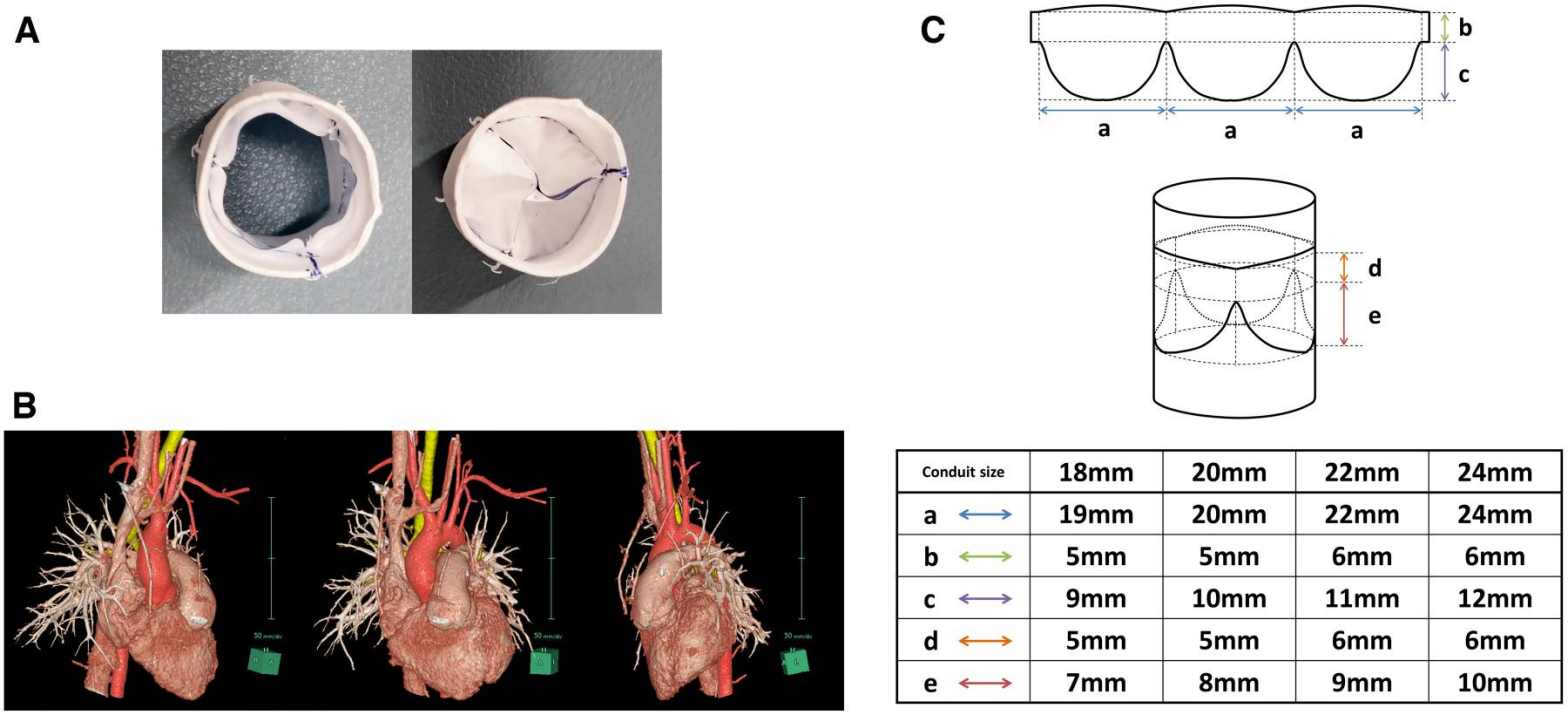
Expanded polytetrafluoroethylene conduit with a hand-sewn tricuspid valve. (**A**) Internal views of the open (left photo) and closed (right photo) position. (**B**) Computed tomography images after 24-mm conduit implantation. (**C**) Design drawing of each conduit size

We also have used ePTFE material for RVOT reconstruction aggressively either in transannular patching or in RV-PA conduit implantation. During transannular patching, a bileaflet valve designed by Nunn is implemented by sewing a 0.1-mm-thick ePTFE membrane below the patch [11]. In patients requiring a small-calibre RV-PA conduit, ePTFE conduits with curved and hand-sewn bileaflet designs are implanted to maintain valve competence even in heterotopic situations as we reported previously [9]. Then, large-calibre ePTFE conduits with a tricuspid valve structurally similar to the normal pulmonary valve are applied in patients whose body size is large enough to eliminate the disadvantages of heterotopic implantation (Fig. 1) [8, 9, 12]. To implant conduits with a diameter as large as possible, we use a standard wall ePTFE tube for the outer frame as it is without creating sinuses. This valved conduit does not need any additional product processing and can be hand-crafted in the same operation room.

The present study reviewed our clinical experience of this ePTFE conduit with a hand-sewn tricuspid valve to analyse its mid- to long-term durability.

## PATIENTS AND METHODS

### Clinical study design

The retrospective study evaluated all patients who received this valved conduit at Kobe Children’s Hospital between May 2010 and December 2022. The institutional review board, or equivalent ethics committee, of the Kobe Children’s Hospital approved the study protocol and publication of the data (R5-110, 1 November 2023). Written consent for the publication of the study data was waived by the institutional review board because of the anonymous use of patient data. Medical records were reviewed, and follow-up was performed by reviewing outpatient charts or contacting the referring physicians. The primary outcomes were survival, catheter intervention for conduit stenosis and conduit replacement. The secondary outcomes were pulmonary valve function, right ventricular function, major haemorrhage, thromboembolic events and infective endocarditis following conduit implantation.

### Valved conduit

This valved conduit with a tricuspid valve was hand-crafted with a standard wall ePTFE tube (diameter, 18–24 mm) and a 0.1-mm thick ePTFE membrane (Video 1) and implanted in a standardized manner (Video 2). Details are provided in Supplementary Material, Appendix S1.

Combined therapy with aspirin and warfarin was maintained for 6–12 months following implantation and discontinued when normal movement of the cusps was confirmed on both echocardiography and cardiac catheterization.

### Haemodynamic evaluation

Pulmonary valve function was evaluated by transthoracic echocardiography using standard views according to guidelines and recommendations [13, 14]. Conduit stenosis was quantified with a peak jet velocity across the valve, and moderate and severe stenosis was defined as a peak velocity of ≥3.0 m/s and ≥4.0 m/s, respectively [13]. Pulmonary regurgitation was graded as none, mild, moderate or severe using the Doppler parameters of the regurgitation jet (size by colour Doppler; density and deceleration rate by continuous wave Doppler) [14]. Right ventricular function was represented with right ventricular end-diastolic volume index (RVEDVI) and right ventricular ejection fraction (RVEF) measured by cardiac catheterization or magnetic resonance imaging. An RVEDVI of ≥160 ml/m^2^ and an RVEF of <40% were considered to indicate severe right ventricular dilation and right ventricular systolic dysfunction, respectively.

### Indication for conduit replacement

Requirements for conduit replacement were based on the current consensus; right ventricular systolic pressure due to conduit stenosis ≥80% of systemic pressure, severe right ventricular dilation or right ventricular systolic dysfunction were mainly adopted for determining the indication [15]. When conduit stenosis was >3.5 m/s or pulmonary regurgitation was greater than moderate on echocardiography, cardiac catheterization was performed to check whether these requirements were met. If conduit stenosis was the dominant aetiology, balloon valvuloplasty was attempted up to two times. Transcatheter pulmonary valve implantation was not performed at our institution because it was not permitted for ePTFE conduits in Japan.

### Statistical analysis

Descriptive statistics were expressed as absolute numbers and percentages of categorical variables. Continuous variables were expressed as mean value ± standard deviation or median with range. Z-scores for pulmonary conduit diameters were calculated using Pettersen *et al*.’s data [16]. Overall survival, freedom from conduit intervention/replacement and freedom from conduit stenosis/regurgitation ≥ moderate were estimated using the Kaplan–Meier method with 95% CI. The correlations of each echocardiographic value with time following conduit implantation, patient’s body weight and patient’s body surface area were analysed by linear regression for each patient, and these results were integrated by linear mixed-effects models in each size of conduits if subjects (the number of patients) were more than 10 [17]. All statistical calculations were performed using the R environment (version 4.0.3; R Foundation, Vienna, Austria).

## RESULTS

### Patient population

Eighty-four consecutive patients were included in this study. Patient characteristics are shown in Table 1 (previous main procedures, Supplementary Material, Table S1). Seventy-three patients had undergone prior surgeries. The indication for conduit implantation in 61 patients was pulmonary valve dysfunction; stenosis, regurgitation, their combination and infective endocarditis were present in 40, 15, 4 and 2 patients, respectively; and two of these patients had left ventricular outflow tract obstruction following physiologic repair of congenitally corrected transposition of the great arteries. In four patients, the right pulmonary artery had been translocated anteriorly during prior surgeries (Lecompte manoeuvre: Rastelli-type, *n* = 2; arterial switch, *n* = 2). Follow-up after discharge was complete. The median follow-up was 4.8 (interquartile range, 2.6–8.0) years. The median echocardiographic follow-up (until reoperation in patients requiring conduit replacement) was 4.7 (interquartile range, 2.5–7.2) years.

**Table 1:** Patient characteristics

Variables	Values (*n* = 84)
Male sex	47 (56)
Age (years)	12 (1.2–40)
Weight (kg)	34 (9.1–82)
Height (cm)	140 (75–178)
Body surface area (m^2^)	1.2 (0.42–2.0)
Initial diagnosis	
Pulmonary atresia + VSD	19 (23)
TOF/DORV + Pulmonary stenosis	18 (21)
Congenital AS/AR	10 (12)
Corrected transposition of the great arteries	9 (11)
Transposition of the great arteries	6 (7)
Pulmonary atresia with intact ventricular septum	5 (6)
CoA/IAA + VSD + AS	4 (5)
Truncus arteriosus	4 (5)
VSD/AVSD + AS	3 (4)
CoA + AS	2 (2)
DORV	2 (2)
Absent pulmonary valve syndrome	2 (2)
Right ventricular function^a^	
Right ventricular end-diastolic volume index (ml/m^2^)	88 (48–170)
Severe right ventricular dilation	2 (2)
Right ventricle ejection fraction (%)	55 (34–82)
Right ventricular systolic dysfunction	3 (4)

Values are presented as *n* (%) or median (range).

AR: aortic regurgitation; AS: aortic stenosis; AVSD: atrioventricular septal defect; CoA: coarctation of the aorta; DORV: double-outlet right ventricle; IAA: interrupted aortic arch; TOF: tetralogy of Fallot; VSD: ventricular septal defect.

a Value calculated with 78 patients’ catheter examinations, in which 4 patients without preoperative catheterization and 2 patients undergoing physiologic repair for corrected transposition of the great arteries were excluded.

### Operative data

The operative details are presented in Table 2 (additional procedures, Supplementary Material, Table S2). Thirty-six conduits were in an orthotopic position, as they were applied in patients undergoing a Rastelli-type operation for tetralogy of Fallot and double-outlet right ventricle without the Lecompte manoeuvre or a Ross operation. The remaining 48 conduits were implanted in a heterotopic position. In five out of six double-switch operations, the hemi-Mustard/bidirectional Glenn procedure was performed for atrial switch. The bidirectional Glenn was also performed concomitantly with pulmonary valve replacement in one patient with pulmonary atresia with intact ventricular septum. In a total of six patients, therefore, the valved conduits served venous blood flow only from the heart and lower body.

**Table 2:** Operative details

Variables	Values (*n* = 84)
Perfusion time (min)	264 (59–585)
Cross-clamp time (min)^a^	164 (26–354)
Type of procedures	
Pulmonary valve/conduit replacement	61 (73)
Ross operation	15 (18)
Double-switch operation	6 (7)
Rastelli-type operation	2 (2)
Conduit size	
18 mm	19 (23)
Patient’s age (years)	4.0 (1.2–12)
Patient’s weight (kg)	14 (9.1–26)
Patient’s body surface area (m^2^)	0.61 (0.42–0.99)
Z-score as the pulmonary valve	1.5 (−0.33–3.1)
20 mm	5 (6)
Patient’s age (years)	6.4 (2.7–12)
Patient’s weight (kg)	18 (14–32)
Patient’s body surface area (m^2^)	0.73 (0.59–1.1)
Z-score as the pulmonary valve	1.5 (0.087–2.4)
22 mm	3 (4)
Patient’s age (years)	9.7 (4.2–22)
Patient’s weight (kg)	20 (15–62)
Patient’s body surface area (m^2^)	0.85 (0.64–1.6)
Z-score as the pulmonary valve	1.5 (−0.38–2.6)
24 mm	57 (67)
Patient’s age (years)	14 (5.8–40)
Patient’s weight (kg)	41 (18–82)
Patient’s body surface area (m^2^)	1.3 (0.72–2.0)
Z-score as the pulmonary valve	0.84 (−0.94–2.8)

Values are presented as median (range) or *n* (%).

a Value calculated with 61 patients, in which 23 patients undergoing conduit implantation on the beating heart were excluded.

### Primary outcomes

**Figure 2:**
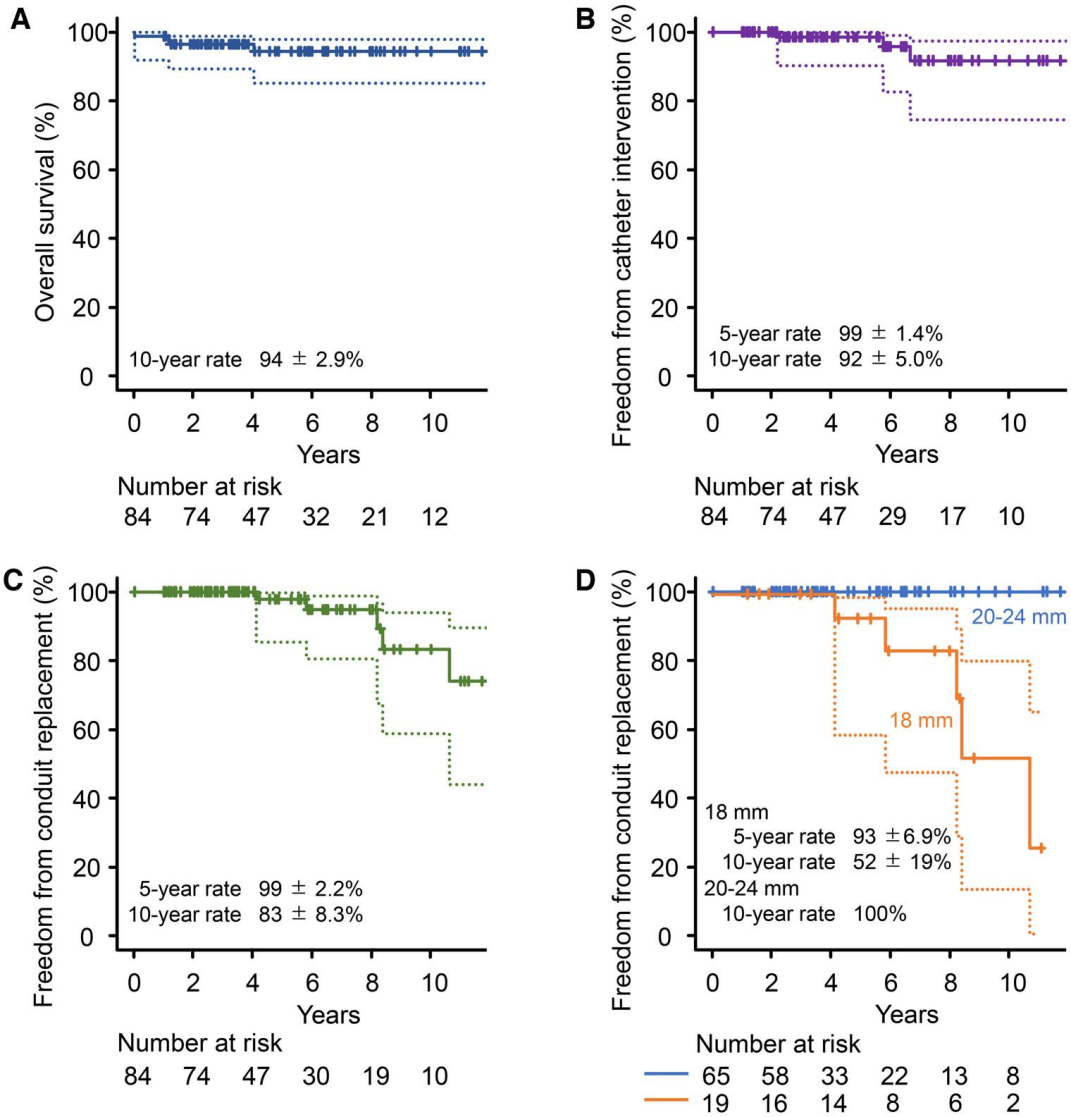
Kaplan–Meier curves for overall survival (**A**), freedom from catheter intervention on the conduit (**B**), freedom from conduit replacement (**C**) and freedom from conduit replacement stratified by conduit size (**D**). Dashed lines denote 95% CI

One patient (1%) died early; a 16-year-old boy requiring aortic and tricuspid valve repair concomitantly with 24-mm conduit implantation, following a Rastelli-type operation for pulmonary atresia with ventricular septal defect, died of sepsis 15 days postoperatively. Three patients (4%) died due to respiratory infection (*n* = 1), neurological complication (*n* = 1) and unknown cause (*n* = 1) during follow-up. The overall survival rate was 94 ± 2.9% (95% CI, 85–98%) at 5 and 10 years (Fig. 2A).

Three patients (4%) required catheter balloon valvuloplasty for conduit stenosis, one of whom required another one; each of 18-, 20- and 24-mm conduits was intervened 5, 6 and 2 years later, respectively. This unexpected early progression of 24-mm conduit stenosis occurred in a 12-year-old girl undergoing pulmonary conduit replacement following truncus arteriosus repair and the Lecompte manoeuvre, due to anterior–posterior compression by the sternum and dilated ascending aorta (Supplementary Material, Fig. S1). Freedom from catheter intervention for conduit stenosis was 99 ± 1.4% (95% CI, 90–100%) and 92 ± 5.0% (95% CI, 75–98%) at 5 and 10 years, respectively (Fig. 2B).

Five patients (6%), all of whom had 18-mm conduits, underwent conduit replacement for conduit stenosis to one 20-, one 22- and three 24-mm ePTFE conduits with the same design. Freedom from conduit replacement was 98 ± 2.2% (95% CI, 85–100%) and 83 ± 8.3% (95% CI, 59–94%) at 5 and 10 years, respectively (Fig. 2C). Three of these conduits were implanted orthotopically during the Ross operation (*n* = 2) or following tetralogy of Fallot repair (*n* = 1) and lasted more than 8 years (Fig. 2D). The remaining two conduits were in a heterotopic position; one with arterial switch operation and atrioventricular groove patch plasty required replacement 5 years later for proximal conduit stenosis, and one with the Yasui operation (*n* = 1) was renewed 4 years later during reoperation for aortic recoarctation (Fig. 2D).

### Secondary outcomes

**Figure 3:**
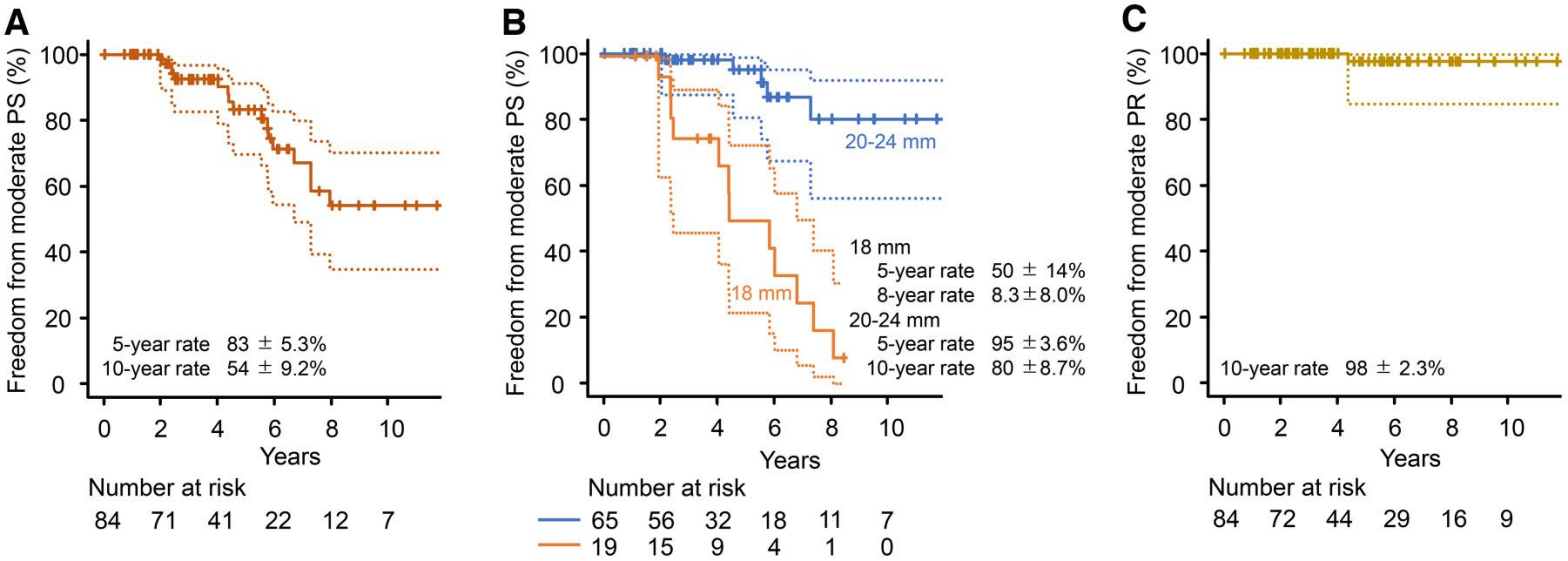
Kaplan–Meier curves for freedom from moderate pulmonary stenosis (PS) (**A**), freedom from moderate PS stratified by conduit size (**B**) and freedom from moderate pulmonary regurgitation (PR) (**C**). Dashed lines denote 95% CI

Freedom from conduit stenosis ≥ moderate was 83 ± 5.3% (95% CI, 70–91%) and 54 ± 9.2% (95% CI, 35–70%) at 5 and 10 years, respectively (Fig. 3A and B for the entire cohort and conduit size stratification, respectively). Freedom from pulmonary regurgitation ≥ moderate was 98 ± 2.3% (95% CI, 85–100%) at 5 and 10 years (Fig. 3C). No moderate or severe pulmonary regurgitation was observed except in one patient with the 18-mm conduit.

**Figure 4:**
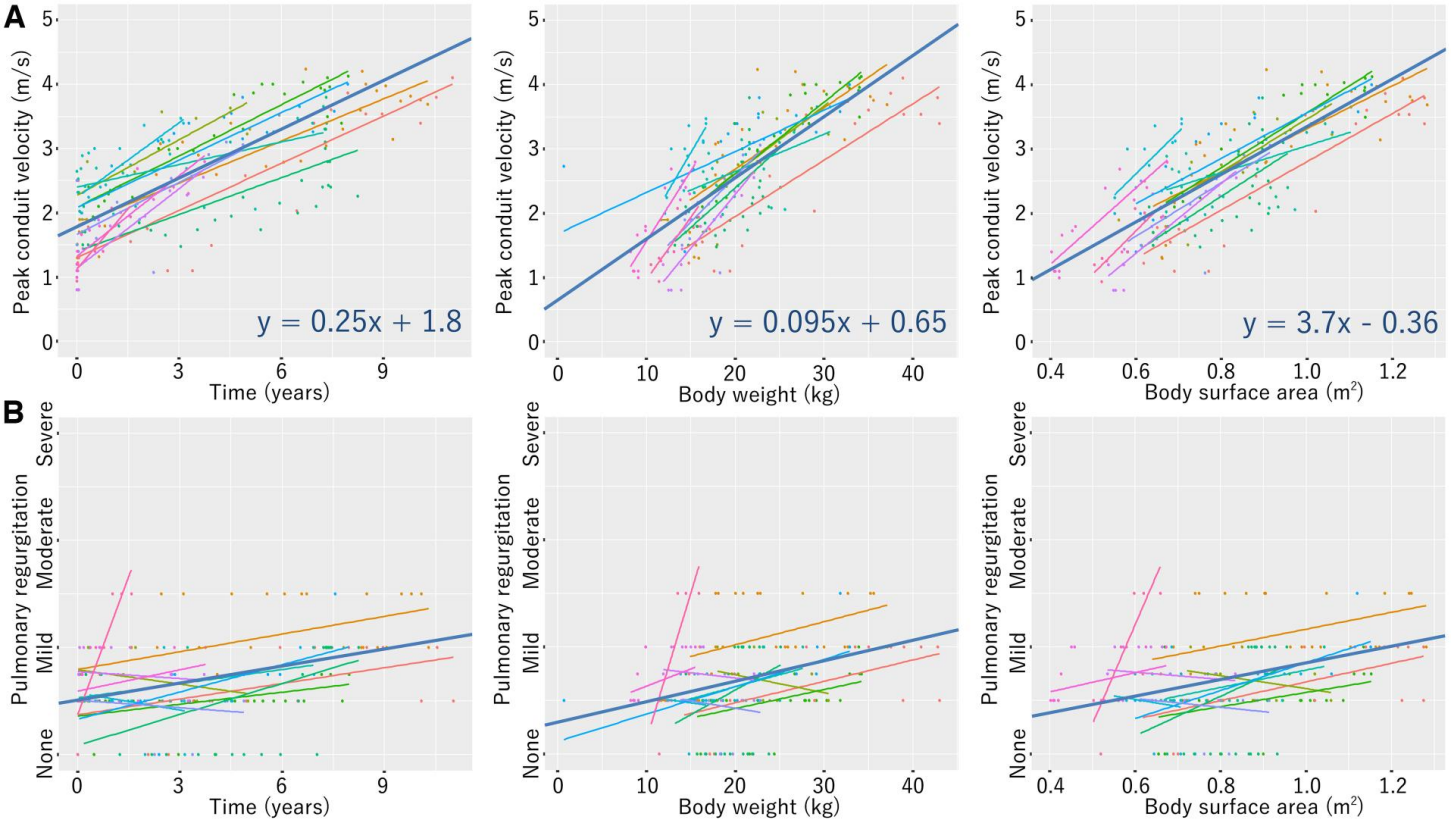
Linear mixed-effects models of echocardiographic peak conduit velocity (**A**) and pulmonary regurgitation (**B**) in patients with the 18-mm conduit

**Figure 5:**
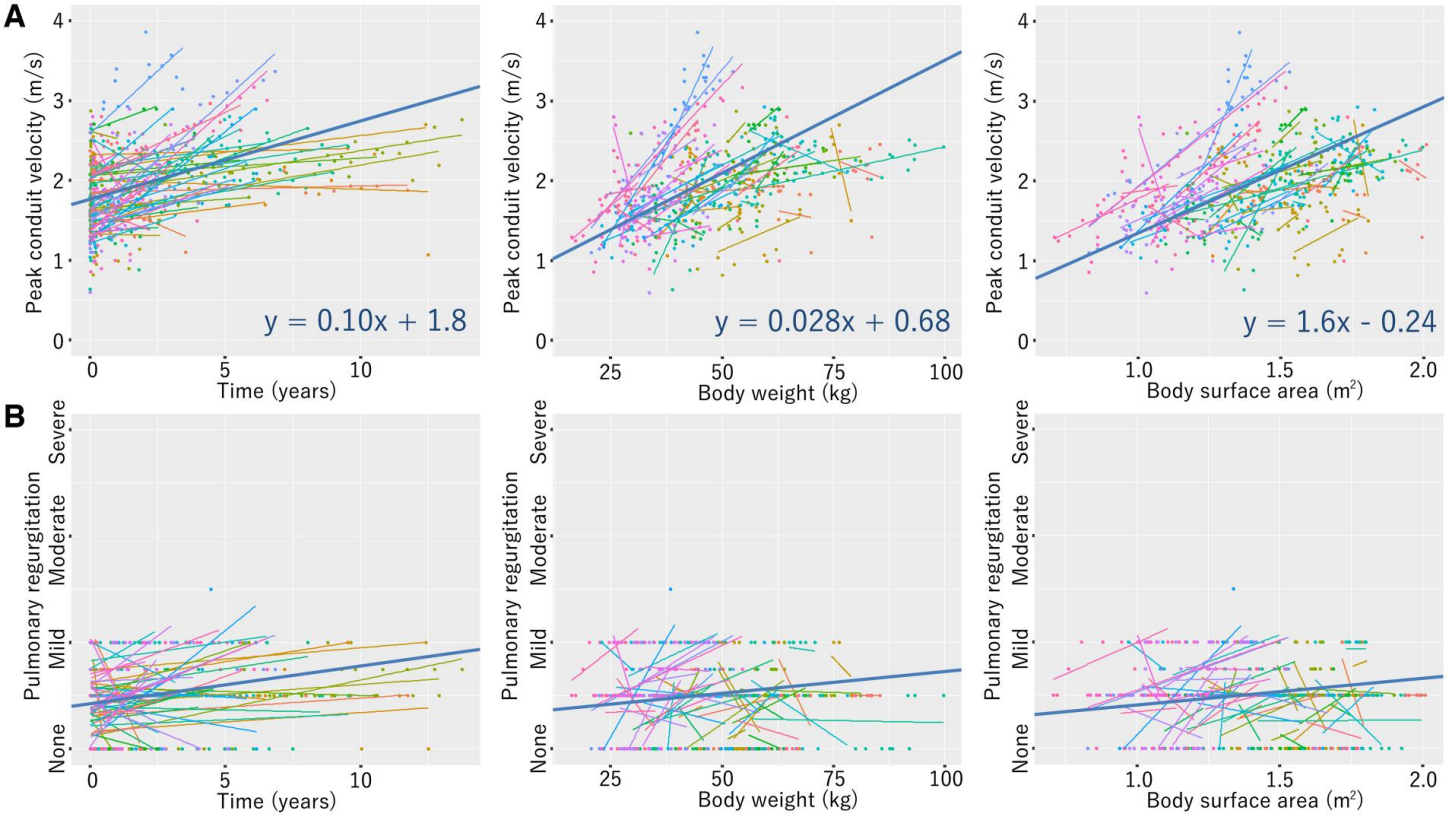
Linear mixed-effects models of echocardiographic peak conduit velocity (**A**) and pulmonary regurgitation (**B**) in patients with the 24-mm conduit

Peak conduit velocity and pulmonary regurgitation were approximated using linear regression models with a total of 895 echocardiographic examinations performed in 75 patients (18-mm conduit, Fig. 4; 20-mm conduit, Supplementary Material, Fig. S2; 22-mm conduit, Supplementary Material, Fig. S3; 24-mm conduit, Fig. 5). One patient who died early, two patients having this conduit in the left ventricular-to-pulmonary artery position and six patients undergoing the bidirectional Glenn procedure were excluded. Linear mixed-effects models were added to the 18- and 24-mm conduit’s regressions.

Postoperative catheterization and magnetic resonance imaging were performed in 56 and 15 patients, respectively. The median RVEDVI and RVEF of the latest examinations were 78 (range, 31–124) ml/m^2^ and 53 (range, 34–82)%. One patient undergoing multiple surgical interventions, including unifocalization and a Rastelli-type operation, had right ventricular systolic dysfunction (RVEF, 34%) with an RVEDVI of 121 ml/m^2^.

Haemorrhage adverse events occurred in one patient; a 34-year-old man with the 24-mm conduit suffered small subdural haematoma 1 month postoperatively but fully recovered. Thromboembolic events and infective endocarditis were detected in one patient; the 18-mm conduit implanted in a 5-year-old boy caused pulmonary valve infective endocarditis and associated pulmonary embolism 7 years postoperatively, both of which were treated medically within the guidelines.

## DISCUSSION

There are an increasing number of patients living with congenital heart disease, and repeated pulmonary valve replacements are often indispensable for patients surviving with diverse RVOT reconstruction [2, 5]. It is obvious that long-lasting pulmonary valve substitutes should contribute to the improved prognosis of these patients. Various options have thus been clinically introduced, and homografts and bioprostheses, including bovine jugular vein conduits and aortic valve prostheses, have gained popularity [1–4]. A systematic review and meta-analysis estimated that homografts and bioprostheses were used in 59% and 41% of grown-up patients’ RVOT reconstruction, respectively, and their overall reintervention rate was 0.72%/y [4]. The mean value of their conduits’ diameter was 26 mm, which is large enough to receive subsequent transcatheter valves to reduce the frequency of reoperation [2, 4].

In the paediatric population, however, their durability becomes significantly shorter even if adult-sized valve prostheses are implantable [1–4]. In patients weighing more than 20 kg from a large paediatric cohort, the 12-year freedom from replacement of pulmonary homografts and composite porcine valves was 72% and 59%, respectively [1]. Another retrospective multicentre study about aortic valve prostheses in the pulmonary position reported that the median time to reintervention in patients <18 years of age was 11 years, and its risk was ∼5 times greater than in patients ≥18 years of age [3]. These early deteriorations are suspected to be immune related and, therefore, the decellularization of biological scaffolds with tissue engineering has been expected to improve the durability and is currently being investigated [18].

On the same theory, ePTFE has also attracted attention as the material for RV-PA valved conduits [6–9]. Although ePTFE cusps are also subject to proteinaceous infiltration and become calcified ultimately [19], their good biocompatibility provides pulmonary conduits with more durable and reliable valve component. In Japanese multicentre studies about ePTFE conduits with bulging sinuses and a fan-shaped valve, freedom from conduit explantation of 20–24 mm conduits implanted at a median age of 14 years was 98% and 91% at 5 and 10 years, respectively [7]. Our 20–24 mm conduits with a hand-sewn tricuspid valve also required no replacement during the study period. These clinical advantages and easy availability of ePTFE facilitate the spread of handmade ePTFE valved conduits to different institutes [12, 20]. Their short- and mid-term outcomes are equivalent to prior works, which indicates that ePTFE valved conduits are a reproducible and predictable solution for RVOT reconstruction.

The present design of our 18–24 mm valved conduits is so intuitive that anyone would come up with it. Thus, almost the same valve design has been applied in several institutes [8, 12]. A relatively large cusp geometric height is created in a straight tube to eliminate the risk of pulmonary regurgitation. It is reasonable that the RVOT should be reconstructed in a fashion like the normal pulmonary valve, where bulging sinuses contribute to the normal cusp movement. In a number of RVOT reconstructions for cardiac anomalies, however, conduits cannot be inserted in such a natural position; irregular anastomotic sites and extracardiac positioning close to the chest wall are not rare circumstances for RV-PA conduits and can mitigate the positive effect of bulging sinuses [4, 9]. We value the actual size of the blood flow pathway and have implanted larger valved conduits than previously described in the literature [7, 12]; roughly, 18-, 20-, 22- and 24-mm conduits are used in patients weighing 10–15 kg, 15–20 kg, 20–25 kg and ≥25 kg, respectively. With these selection criteria, any conduit does not exceed a Z-score of 3 as the pulmonary valve and can be placed without technical difficulty [16]. Once the 24-mm conduit is implanted, it is supported to function with a peak velocity <3.0 m/s and without moderate/severe regurgitation in patients with a body weight of up to 75 kg and a body surface area of up to 2.0 m^2^ for more than 12 years postoperatively, based on the linear mixed-effects models. The same models also implied the 18-mm conduit can be durable for 8 years until the patient’s weight reaches 30 kg. We believe that our approaches led to these favourable clinical outcomes.

Nevertheless, it is necessary to pay attention to the disadvantages of heterotopic implantation [4, 9, 21]. In this cohort, all three conduits (18-mm conduit, *n* = 2; 24-conduit, *n* = 1) requiring unexpected early reintervention had been placed heterotopically. The failure of the 24-mm conduit was due to compression with surrounding structures and a typical example of adverse events with heterotopic implantation. Following this experience, the anterior V-shaped reconstruction with two 18-mm conduits is aggressively applied in patients with the dilated ascending aorta and the anterior reconstructed RVOT to avoid the same situation (Supplementary Material, Fig. S4) [9]. A diameter of 18 mm is large enough for one of the branch pulmonary arteries to serve sufficient pulmonary blood flow, even in grown-up patients [22].

Meanwhile, these are several drawbacks of these valved conduits. Because the product lineup of the standard wall ePTFE tube is composed of 12–24 mm, the 24-mm conduit is the only choice for patients weighing ≥25 kg. Even in patients weighing 90 kg, however, a diameter of 24 mm is still a Z-score of −2.5 as pulmonary valve diameter [16], and our patients weighing 75–100 kg are doing well with a peak conduit velocity of < 3.0 m/s. Moreover, postoperative antiplatelet and anticoagulant therapies for 6–12 months remain controversial for ePTFE conduits and may have triggered postoperative subdural haematoma in our adult patient [7, 12]. While thromboembolic risk should be managed in a prudent manner, especially for products with new designs, it might be better to apply only anticoagulant therapy for adult patients in the same way as established with bioprosthetic valve implantation. Finally, both experience and literature of transcatheter pulmonary valve implantation for failed ePTFE conduits are still quite limited [20]. At least transcatheter valves with an outer diameter >24 mm cannot be inserted because ePTFE conduits are not dilated. Patients ≥18 years of age whose body size is large enough to receive valve substitutes with an inner diameter ≥27 mm may benefit more from the use of homografts or bioprostheses and subsequent transcatheter valve implantation.

### Limitations

The clinical study design had limitations inherent to a single-centre retrospective follow-up study that enrolled a small number of patients over a long period. Only a small number of patients were followed up for >10 years. Magnetic resonance imaging for accurate assessment of pulmonary regurgitation is lacking. The number of patients with 20- and 22-mm conduits was insufficient for linear mixed-effects models. No comparison with surgical options such as homografts and bioprostheses was performed.

## CONCLUSION

Our ePTFE conduit with a hand-sewn tricuspid valve has shown favourable clinical outcomes with durable valve competence and low reintervention rates. This valved conduit is a valid alternative to homografts and bioprostheses, especially in young patients with increased risk for early failure of the existing products.

## Supplementary Material

ivaf020_Supplementary_Data

## Data Availability

The data underlying this article will be shared on reasonable request to the corresponding author.

## Notes

Presented at the 104th Annual Meeting of The American Association for Thoracic Surgery, Toronto, Canada, April 27–30, 2024.

## ABBREVIATIONS

RVOT: Right ventricular outflow tract
RV-PA: Right ventricle-to-pulmonary artery
ePTFE: Expanded polytetrafluoroethylene
RVEDVI: Right ventricular end-diastolic volume index
RVEF: Right ventricular ejection fraction

## References

[1] Willetts RG, Stickley J, Drury NE et al Four right ventricle to pulmonary artery conduit types. J Thorac Cardiovasc Surg 2021;162:1324–33.e3. 33640135 10.1016/j.jtcvs.2020.12.144

[2] Boethig D, Avsar M, Bauer UMM et al; National Register For Congenital Heart Defects Investigators. Pulmonary valve prostheses: patient’s lifetime procedure load and durability. Evaluation of the German National Register for Congenital Heart Defects. Interact CardioVasc Thorac Surg 2022;34:297–306. 34436589 10.1093/icvts/ivab233 PMC8929479

[3] Baird CW, Chávez M, Sleeper LA et al Reintervention rates after bioprosthetic pulmonary valve replacement in patients younger than 30 years of age: a multicenter analysis. J Thorac Cardiovasc Surg 2021;161:345–62.e2. 33069421 10.1016/j.jtcvs.2020.06.157

[4] Wang X, Bakhuis W, Veen KM et al Outcomes after right ventricular outflow tract reconstruction with valve substitutes: a systematic review and meta-analysis. Front Cardiovasc Med 2022;9:897946. 36158811 10.3389/fcvm.2022.897946 PMC9489846

[5] Holst KA, Dearani JA, Burkhart HM et al Risk factors and early outcomes of multiple reoperations in adults with congenital heart disease. Ann Thorac Surg 2011;92:122–8. 21718837 10.1016/j.athoracsur.2011.03.102

[6] Yamagishi M. Right ventricular outflow reconstruction using a polytetrafluoroethylene conduit with bulging sinuses and tricuspid fan-shaped polytetrafluoroethylene valve. Oper Tech Thorac Cardiovasc Surg 2016;21:211–29.

[7] Hongu H, Yamagishi M, Maeda Y et al Expanded polytetrafluoroethylene conduits with bulging sinuses and a fan-shaped valve in right ventricular outflow tract reconstruction. Semin Thorac Cardiovasc Surg 2022;34:972–80. 33691193 10.1053/j.semtcvs.2021.02.026

[8] Ootaki Y, Welch AS, Walsh MJ, Quartermain MD, Williams DA, Ungerleider RM. Medium-term outcomes after implantation of expanded polytetrafluoroethylene valved conduit. Ann Thorac Surg 2018;105:843–50. 29100642 10.1016/j.athoracsur.2017.07.013

[9] Matsushima S, Matsuhisa H, Wakita K et al Expanded polytetrafluoroethylene conduits with curved and handsewn bileaflet designs for right ventricular outflow tract reconstruction. J Thorac Cardiovasc Surg 2024;167:439–49.e6. 37356475 10.1016/j.jtcvs.2023.05.043

[10] Baird CW, Chávez M, Backer CL, Galantowicz ME, Del Nido PJ. Preliminary results with a novel expanded polytetrafluoroethylene-based pulmonary valved conduit. Ann Thorac Surg 2022;114:2314–21. 34838744 10.1016/j.athoracsur.2021.10.033

[11] Nunn GR, Bennetts J, Onikul E. Durability of hand-sewn valves in the right ventricular outlet. J Thorac Cardiovasc Surg 2008;136:290–6. 18692631 10.1016/j.jtcvs.2008.02.063

[12] Shi Q, Shan Y, Chen G et al Midterm outcomes for polytetrafluoroethylene valved conduits. Ann Thorac Surg 2022;114:1778–85. 34717907 10.1016/j.athoracsur.2021.09.051

[13] Baumgartner H, Hung J, Bermejo J et al; European Association of Echocardiography. Echocardiographic assessment of valve stenosis: EAE/ASE recommendations for clinical practice. J Am Soc Echocardiogr 2009;22:1–23. 19130998 10.1016/j.echo.2008.11.029

[14] Zoghbi WA, Enriquez-Sarano M, Foster E et al; American Society of Echocardiography. Recommendations for evaluation of the severity of native valvular regurgitation with two-dimensional and Doppler echocardiography. J Am Soc Echocardiogr 2003;16:777–802. 12835667 10.1016/S0894-7317(03)00335-3

[15] Stout KK, Daniels CJ, Aboulhosn JA et al 2018 AHA/ACC Guideline for the Management of Adults With Congenital Heart Disease: a report of the American College of Cardiology/American Heart Association Task Force on Clinical Practice Guidelines. Circulation 2019;139:e637–e97. 30586768 10.1161/CIR.0000000000000602

[16] Pettersen MD, Du W, Skeens ME, Humes RA. Regression equations for calculation of z scores of cardiac structures in a large cohort of healthy infants, children, and adolescents: an echocardiographic study. J Am Soc Echocardiogr 2008;21:922–34. 18406572 10.1016/j.echo.2008.02.006

[17] Wang X, Andrinopoulou ER, Veen KM, Bogers AJJC, Takkenberg JJM. Statistical primer: an introduction to the application of linear mixed-effects models in cardiothoracic surgery outcomes research—a case study using homograft pulmonary valve replacement data. Eur J Cardiothorac Surg 2022;62:ezac429. 36005884 10.1093/ejcts/ezac429 PMC9496250

[18] Chauvette V, Bouhout I, Tarabzoni M et al; Canadian Ross Registry. Pulmonary homograft dysfunction after the Ross procedure using decellularized homografts-a multicenter study. J Thorac Cardiovasc Surg 2022;163:1296–305.e3. 32888704 10.1016/j.jtcvs.2020.06.139

[19] Yamamoto Y, Yamagishi M, Maeda Y et al Histopathologic analysis of explanted polytetrafluoroethylene-valved pulmonary conduits. Semin Thorac Cardiovasc Surg 2020;32:990–9. 31606427 10.1053/j.semtcvs.2019.10.001

[20] Diaz-Castrillon CE, Castro-Medina M, Viegas M et al Anatomic position and durability of polytetrafluoroethylene conduit ≥18 mm: single-center experience. Ann Thorac Surg 2023;115:983–9. 35988739 10.1016/j.athoracsur.2022.08.013

[21] Brown JW, Ruzmetov M, Rodefeld MD, Vijay P, Turrentine MW. Right ventricular outflow tract reconstruction with an allograft conduit in non-ross patients: risk factors for allograft dysfunction and failure. Ann Thorac Surg 2005;80:655–63. 16039222 10.1016/j.athoracsur.2005.02.053

[22] Burman ED, Keegan J, Kilner PJ. Pulmonary artery diameters, cross sectional areas and area changes measured by cine cardiovascular magnetic resonance in healthy volunteers. J Cardiovasc Magn Reson 2016;18:12. 26940894 10.1186/s12968-016-0230-9 PMC4778312

