# Supplementary material for: Pulmonary expanded polytetrafluoroethylene conduits with a hand-sewn tricuspid valve
Source: Interdiscip Cardiovasc Thorac Surg. 2025 Feb 6;40(2):ivaf020. doi: 10.1093/icvts/ivaf020 (PMC11997764; doi:10.1093/icvts/ivaf020)
Supplement: ivaf020_Supplementary_Data [file ivaf020_Supplementary_Data.zip › Supplementary_Material_Apendix_S1.docx]

**Valved conduit design**

A 0.1-mm thick ePTFE membrane and a standard wall ePTFE tube (GoreTex; W.L. Gore & Associates, Flagstaff, Ariz) were used for the cusp and conduit material, respectively (Video 1). The tube diameters ranged from 18 mm to 24 mm in 2-mm increments, and details of cusp component design were determined according to conduit size and presented in Fig. 1C. A piece of connected cusps was cut out from the ePTFE membrane. The ePTFE tube was inverted, and commissural and cusp insertion lines were drawn on the inner wall. The piece of cusps was sewn to the inner wall from commissural posts to cusp nadirs along the marked lines. Each top of commissural lines was reinforced with an additional stitch. The ePTFE tube was again inverted. A single suture was placed on the cusp free margins near each of the commissural posts, creating slight partial cusp fusion to prevent the cusp from sticking to the conduit wall. ePTFE sutures (GoreTex CV-6; W.L. Gore & Associates, Flagstaff, Ariz) were used for each stitching.

**Surgical technique**

Surgery was performed through median sternotomy with cardiopulmonary bypass (Video 2). The branch pulmonary arteries were frequently augmented with autologous pericardium or cutout ePTFE patches to relieve peripheral pulmonary stenosis and enlarge the pulmonary bifurcation to the conduit caliber. The distal end of the valved conduit was trimmed slightly obliquely to shorten its lesser curvature, on which any one of the commissural posts was located, and anastomosed to the pulmonary bifurcation using a continuous CV-6 suture.

A U-shaped excision was made on the lesser curvature of the proximal end to create a low-profile dorsal side of the valved conduit. This excision should often be extended near the intercusp triangle below the posterior commissure. The dorsal bottom was anastomosed to the upper edge of the right ventriculotomy by using a continuous 5-0 polypropylene suture (Prolene; Ethicon Inc., Somerville, NJ). The proximal end of the valved conduit was further trimmed along the remaining edge of the right ventriculotomy, in which the great curvature was left on the long side to create a proximal hood. The proximal anastomosis was completed using the continuous 5-0 polypropylene suture.
