# Supplementary material for: Pulmonary expanded polytetrafluoroethylene conduits with a hand-sewn tricuspid valve
Source: Interdiscip Cardiovasc Thorac Surg. 2025 Feb 6;40(2):ivaf020. doi: 10.1093/icvts/ivaf020 (PMC11997764; doi:10.1093/icvts/ivaf020)
Supplement: ivaf020_Supplementary_Data [file ivaf020_Supplementary_Data.zip › Supplementary_Material_Figure_legends.docx]

**Supplementary Material, Figure legends**

**Figure S1:** Preoperative computed tomography image (A) and postoperative catheterization (B) of the patient with unexpected early progression of 24-mm conduit stenosis. The conduit was deformed with anterior-posterior compression by the sternum and dilated ascending aorta.

**Figure S2:** Linear regression models of echocardiographic peak conduit velocity (A) and pulmonary regurgitation (B) in patients with the 20-mm conduit.

**Figure S3:** Linear regression models of echocardiographic peak conduit velocity (A) and pulmonary regurgitation (B) in patients with the 22-mm conduit.

**Figure S4:** Anterior V-shaped reconstruction with 2 18-mm conduits in a 12-year-old boy with truncus arteriosus repair (A) and a 13-year-old boy with the Yasui operation (B).
