# Supplementary material for: Pulmonary expanded polytetrafluoroethylene conduits with a hand-sewn tricuspid valve
Source: Interdiscip Cardiovasc Thorac Surg. 2025 Feb 6;40(2):ivaf020. doi: 10.1093/icvts/ivaf020 (PMC11997764; doi:10.1093/icvts/ivaf020)
Supplement: ivaf020_Supplementary_Data [file ivaf020_Supplementary_Data.zip › Supplementary_Material_Tables.docx]

**Table S1:** Previous main procedures

| Variables | Values (n=84) |
| --- | --- |
| Rastelli-type operation | 27 (32) |
| TOF/DORV ± APVS repair | 20 (24) |
| Percutaneous aortic valvulotomy | 5 (6) |
| Aortic valvuloplasty | 4 (5) |
| Subaortic stenosis repair | 3 (4) |
| Yasui operation | 3 (4) |
| Palliative right ventricle-to-pulmonary artery shunt | 3 (4) |
| Pulmonary atresia with intact ventricular septum repair | 3 (4) |
| Percutaneous pulmonary valvulotomy | 2 (2) |
| Arterial switch operation | 2 (2) |
| Physiologic repair of cTGA | 2 (2) |
| Pulmonary artery banding | 2 (2) |
| Ross operation | 1 (1) |
| Bidirectional Glenn operation | 1 (1) |
| Double-switch operation | 1 (1) |
| None | 5 (6) |

Values are presented as n (%). APVS, Absent pulmonary valve syndrome; cTGA, corrected transposition of the great arteries; DORV, double-outlet right ventricle; TOF, tetralogy of Fallot.

**Table S2:** Additional procedures with conduit implantation

| Variables | Values |
| --- | --- |
| Pulmonary artery plasty | 47 (56) |
| Right | 9 (11) |
| Left | 14 (17) |
| Bilateral | 24 (28) |
| Tricuspid valve repair | 4 (5) |
| Aortic valve repair/replacement | 3 (4) |
| Repair of left ventricular outflow tract obstruction | 3 (4) |
| Repair of aberrant subclavian artery | 3 (4) |
| Closure of intracardiac shunt | 3 (4) |
| Anterior translocation of the right pulmonary artery | 2 (2) |
| Warden operation | 2 (2) |
| Pacemaker implantation | 2 (2) |
| Ablation | 2 (2) |
| Bidirectional Glenn operation | 1 (1) |
| Mitral valve repair | 1 (1) |
| Coronary artery bypass grafting | 1 (1) |

Values are presented as n (%).
